# A 3D-printed portable device for field deployment of living biosensors

**DOI:** 10.1016/j.mex.2019.05.018

**Published:** 2019-05-28

**Authors:** Paul G. Movizzo, Warren C. Ruder, Zhicheng Long

**Affiliations:** Department of Bioengineering, University of Pittsburgh, United States

**Keywords:** 3D-printed containment of living biosensors, Synthetic biology, Additive manufacturing, Biosensors

## Abstract

Living biosensors typically use genetically modified organisms (GMOs) to detect infectious biomarkers in clinical samples. GMOs are prohibited to be released into the environment by many laws, which limits the application of living biosensors outside laboratory settings. Here, we reported a robust 3D-printed device that eliminates the risk of exposure of GMOs to potential users and the environment. The device is designed to snugly attach to a common culture tube and consists of two components, a housing and a plunger. The housing contains a stress-focusing cutout and a reagent well to hold the living biosensor. While the plunger is designed to form a two-stage press-fit seal with the housing. The first seal allows to safely transfer the living biosensor from a biosafety lab to the field, and the second seal prevents the leakage of GMOs from the culture tube during the test and before safe disposal. Additionally, a lever-actuated machine was also designed and 3D-printed to assist users operating the device.

## Graphical abstract

**Figure:**
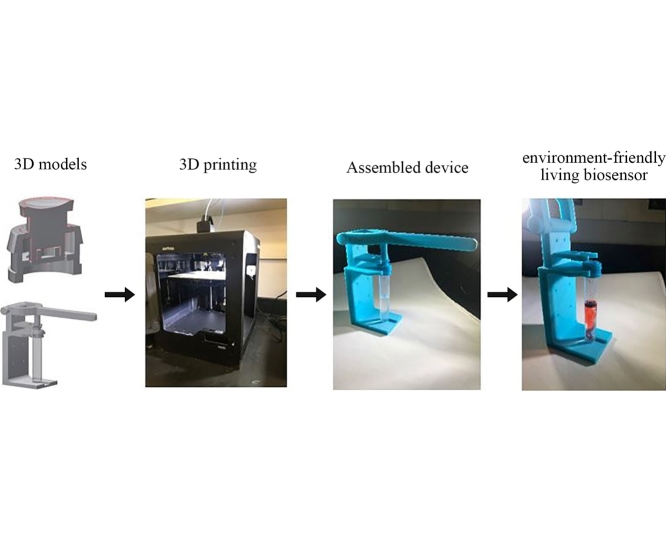

**Specifications Table**

**Table:** 

Subject Area:	*Engineering*
More specific subject area:	*Bioengineering*
Method name:	*3D-Printed Containment of Living Biosensors*
Name and reference of original method:	*See companion Engineering Research article.*
Resource availability:	*3D-printed parts files available*

## Method details

**Fig. 1:**
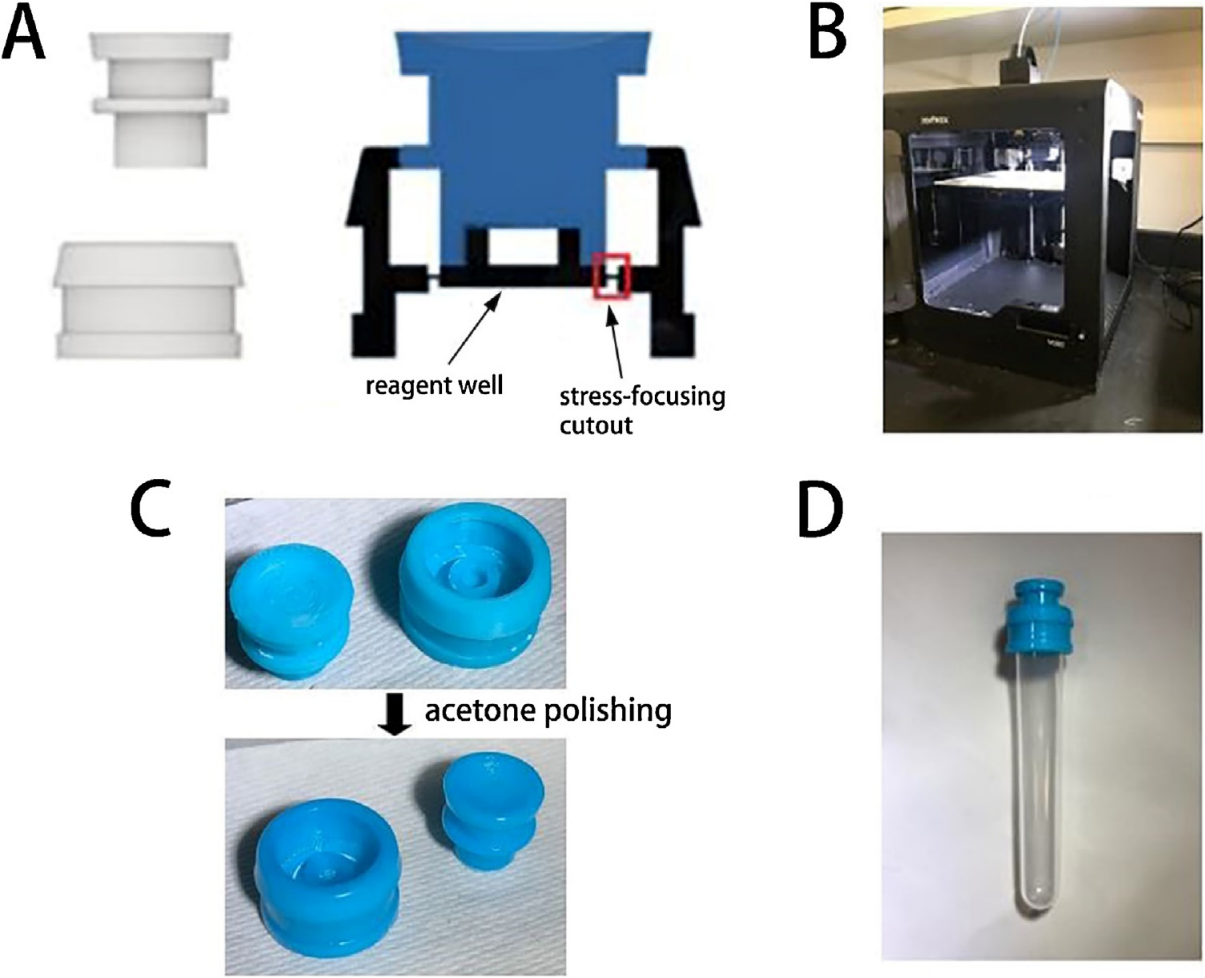
Fabrication of the environment-friendly bioassay device. (A) 3D designs of the device including labeled features. (B) The designs were 3D-printed with Z-ABS material using a Zortrax® M200 3D printer. (C) The 3D-printed housing and plunger before and after exposure to an acetone vapor bath for 1 h. (D) The fully assembled bioassay device attached to a 14 mL culture tube. Note that the first press-fit seal is formed.

As shown in Fig. 1, the device consists of 2 separate parts; a housing and a plunger, which were 3D-printed independently. The device was primarily designed to isolate the potentially biohazardous living cells used in living biosensors from the environment during the entire biosensing process. This isolation is achieved through the two-stage press-fit seal between the housing and the plunger, as well as the water tight seal between the housing and the culture tube.

The device was originally developed to be used by simply pressing an individual person’s thumb on the plunger [1]. Here, we expanded upon the original technique by also developing an assistive lever-actuated machine to both ensure that all scientists and non-technical users will be able to break the cutout of the housing to release the living biosensor into the sample tube, but also so that a wide range of strong plastic materials can be used effectively when 3D-printing this device. Both the bioassay device and the lever-actuated machine were designed using Autodesk® Inventor Professional 2018 and were printed with Z-ABS material on a Zortrax® M200 3D-printer, an affordable and widely available model. The 3D design files of the bioassay device and lever-actuated machine are available for download as supplementary files.

### Development of the environment-friendly bioassay device

1 To accommodate the use of potentially dangerous biological substances in the field, the plunger and housing were designed to create a press-fit seal when they are first put together (Fig. 1A). This seal allows the living cells enclosed in the device to be safely transferred from a biosafety laboratory to the testing field.
2 In the field, A VWR® 14 mL culture tube containing a test sample can then be attached to the bottom of the housing which creates a water tight seal. The biosensing process can then be activated by applying pressure to the top of the plunger which breaks the stress-focusing cutout and releases the reagent well and the living cells into the culture tube.
3 The second stage of the seal between the housing and the plunger was designed to permanently close the device and the test tube, which ensures the isolation of the biohazardous waste from the environment. The entire device can then be transferred back to a biosafety laboratory for proper disposal (e.g., by autoclave, or if necessary, by disassembly and treatment with bleach).
4 To smooth the surface of the 3D-printed device and create better seal, the ABS-based devices were subjected to an acetone vapor bath. This was performed by first placing printed housings and plungers on aluminum foil in an airtight glass container. Then, paper towels were soaked with acetone and placed in the airtight glass container and were held in place by the weight of the container lid. The container was closed, and the device was kept in the container for 1 h, after which the parts were exposed to ambient air for an additional 2 h (Fig. 1C).

### Development of the lever-actuated machine

**Fig. 2:**
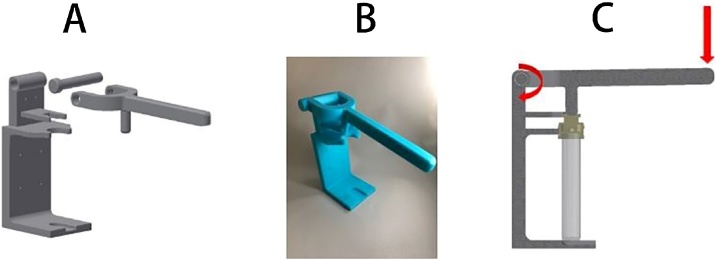
Development and use of the lever-actuated machine. (A) The 3D design of the lever-actuated machine. (B) The assembled 3D-printed lever-actuated machine ready for use. (C) A cross-sectional diagram showing that application of force to the end of the lever results in rotation of the lever around a fixed axis and a pin on the lever contacts the bioassay device, forcing the plunger into the housing.

5 A custom lever-actuated machine was developed to enable a wide range of 3D printing materials to be used for the device and to allow the bioassay device to be easily operated by personnel with varying grip strength. The machine was designed as a simple lever-actuated mechanism that allows the entire bioassay device (including the culture tube) to be secured to a platform to prevent the device from slipping during actuation (Fig. 2). As force is applied to the lever arm, it rotates around a fixed axis and a pin to make contact with the plunger, forcing the plunger into the housing to break the stress-focusing cutout and to create the press-fit seals. This machine was designed to be wall-mounted or simply used on a flat surface. Due to the extra downward force that this machine can provide, a 3D-printed bioassay device may be created using materials with a high elastic modulus and will still function as intended.

## Method validation

### Testing the press-fit seals for leak test with dye

**Fig. 3:**
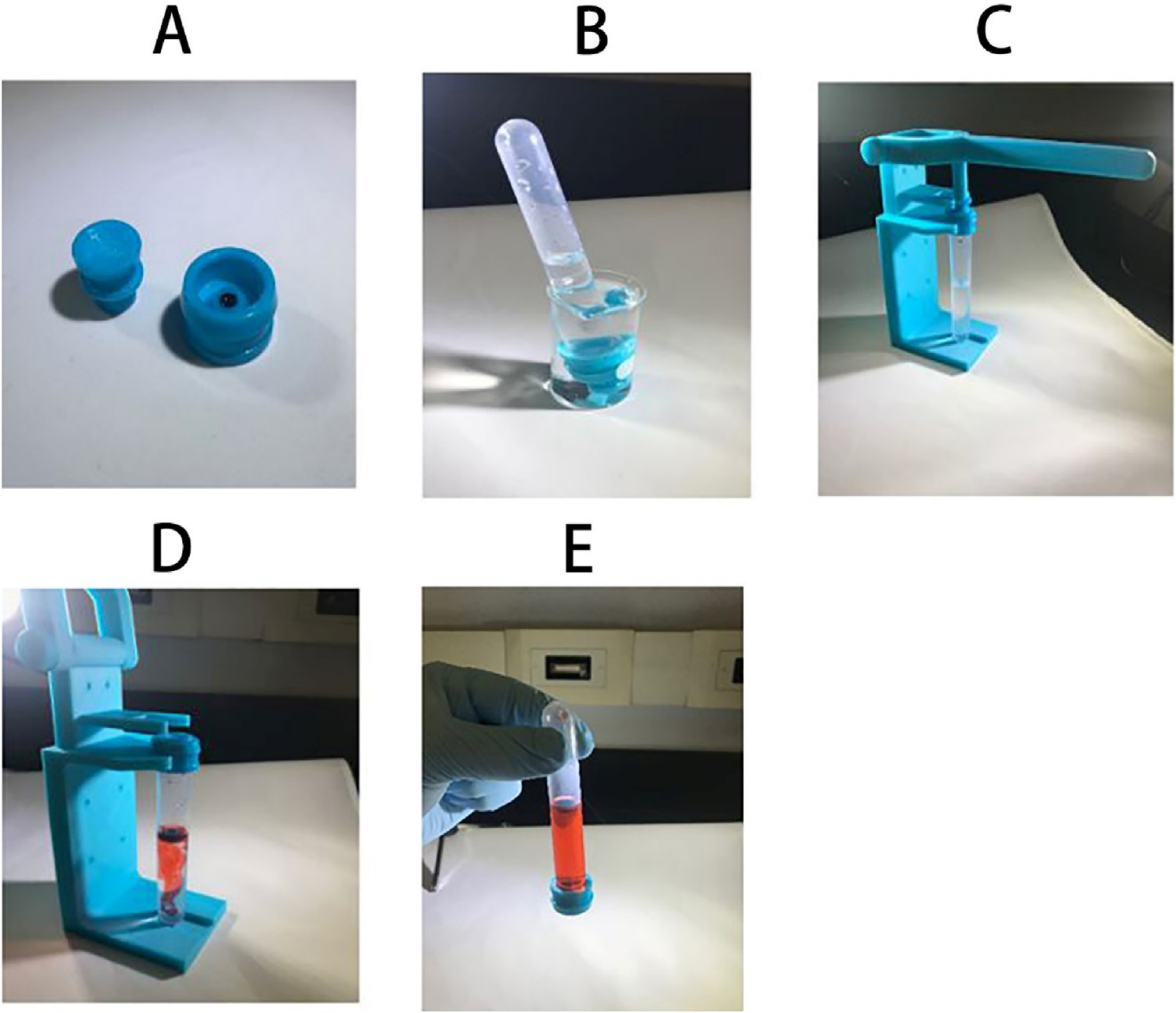
Device operation and leak test. (A) A newly 3D-printed housing with a well filled with 50 μL of red (food) dye. (B) A housing and plunger were assembled to form the first stage of the press-fit seal, then attached to water-filled test tube and entire device was inverted and submerged into a water-filled beaker for 12 h to test for leaks. This step is not necessary for normal device operation. (C) The device was removed from the beaker and dried, then attached to a lever-actuated machine. (D) The stress-focusing cutout was broken by the lever-actuated machine and the reagent well containing red dye dropped into the test tube. The second stage of the seal was also formed to create an isolated system. (E) The sealed device was leak free and could be safely analyzed and transported.

To mimic the use of the 3D-printed device in the field, we used a red-dyed water as a surrogate for the genetically engineered cells used in living biosensors. We first loaded 50 μL of liquid red (food) dye in the reagent well in the housing (Fig. 3A). A plunger was then pressed into the housing to create the first stage of the press-fit seal. A test tube filled with water was also attached to the housing and this entire device was inverted and submerged into a water-filled beaker for 12 h. If the stress-focusing cutout or press-fit seal were to leak, water in the test tube or beaker, respectively, would turn a red color. After this 12 h period, no red dye was observed to have escaped from the housing (Fig. 3B), suggesting the first stage of the press-fit seal on the device could reliably prevent the leakage of living cells to an aqueous environment during transport. The device was then placed in the lever-actuated machine and the cutout on the housing failed, causing the reagent well to drop into the test tube and flood the water inside the test tube with red dye (Fig. 3D). When the second stage of press-fit seal was created, the test tube with the 3D-printed device was again inverted and submerged into a beaker filled with water. No dyed water was observed in the beaker after 12 h.
